# The invasive front in hepatocellular carcinoma: toward a tumor–CAF–macrophage metabolic interface

**DOI:** 10.3389/fimmu.2026.1840563

**Published:** 2026-05-29

**Authors:** Dan Wei, Wenzhi Guo, Wei Dong

**Affiliations:** 1 Department of Critical Care Medicine, The First Affiliated Hospital of Zhengzhou University, Zhengzhou, Henan, China; 2 Department of Hepatobiliary and Pancreatic Surgery, The First Affiliated Hospital of Zhengzhou University, Zhengzhou, Henan, China

**Keywords:** cancer-associated fibroblasts, hepatocellular carcinoma, invasive front, macrophages, metabolic interface, spatial multi-omics

## Introduction

Hepatocellular carcinoma (HCC) is both a metabolically reprogrammed malignancy and a spatially heterogeneous immune microenvironment. Existing precision-oncology frameworks have greatly improved the classification of malignant, stromal, and immune cell states in HCC, while more recent spatial multi-omics studies suggest that progression-associated biology is often concentrated at the invasive front (1–4). These observations suggest that spatial organization can extend cell-state-based models of HCC by placing cellular programs back into their immediate tissue context (4, 5).

Several recent studies support this view. Recent HCC studies collectively suggest that the invasive front contains spatially restricted regions characterized by local metabolic change, stromal organization, and immune accessibility, further supporting the biological relevance of interface-based interpretation (1–4).

**Figure 1:**
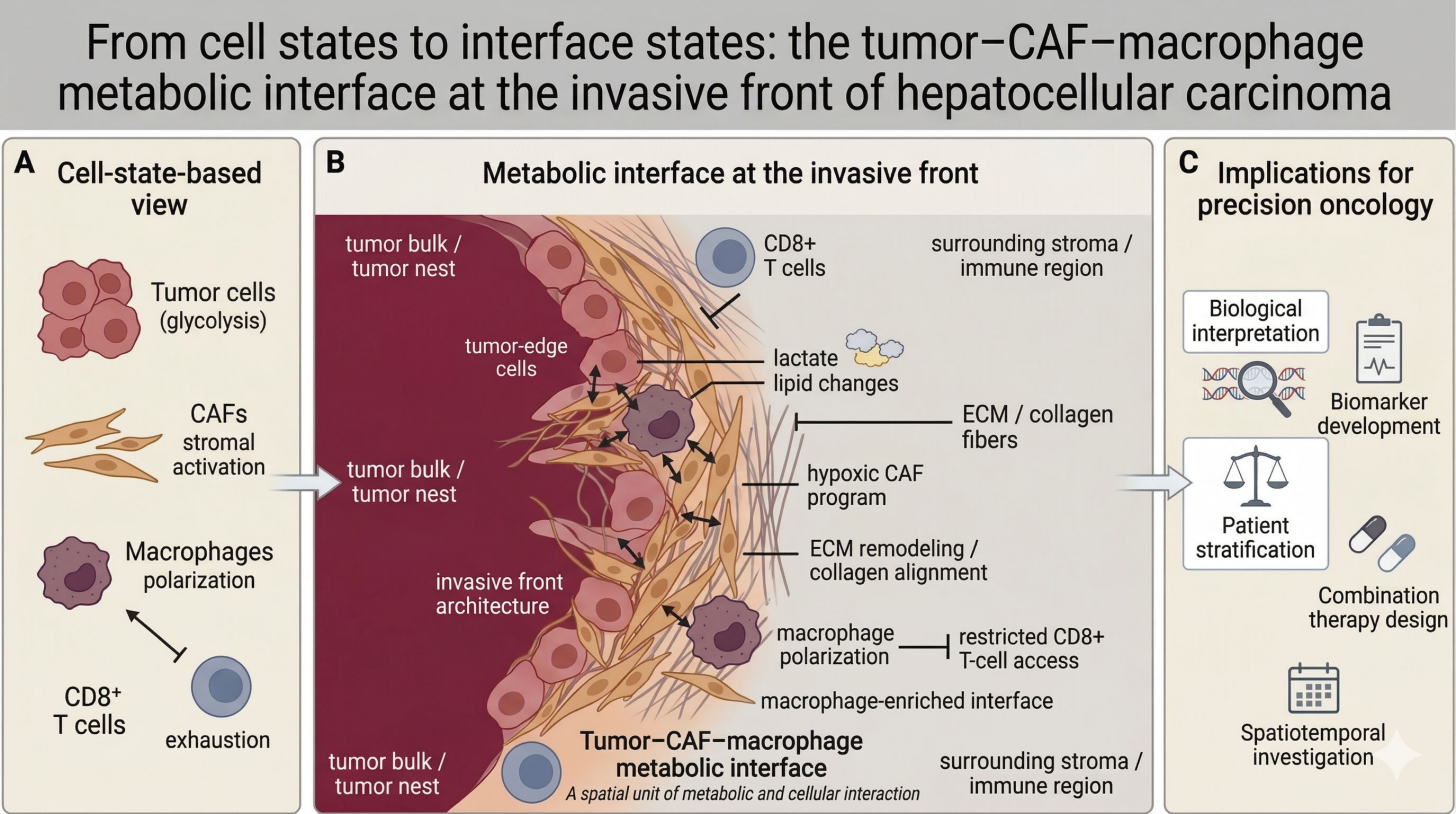
Conceptual model of the tumor–CAF–macrophage metabolic interface at the invasive front of hepatocellular carcinoma. **(A)** The conventional cell-state-based view of the tumor microenvironment. **(B)** The invasive front may be viewed as a spatially restricted metabolic interface, where tumor-edge cells, CAFs, macrophages, ECM/collagen fibers, and lymphocytes coexist under shared metabolic and structural conditions. This interface is associated with local metabolic changes, stromal remodeling, macrophage polarization, and restricted CD8^+^ T-cell access. **(C)** The potential relevance of this framework to biological interpretation, biomarker development, patient stratification, combination-therapy design, and spatiotemporal investigation.

In this opinion, we suggest that the HCC invasive front is especially informative when interpreted as a tumor–CAF–macrophage metabolic interface, in which metabolic, stromal, and immune processes are locally integrated (**Figure 1**). Borrowing conceptually from Su et al., who described a leading-edge multicellular ecosystem (LEMCE) in head and neck squamous cell carcinoma (HNSCC), we propose that a related spatial framework may also be useful in HCC (6). Here, the metabolic interface denotes invasive-front regions where metabolic, stromal, and immune features converge within the same local tissue environment. Our central argument is not to replace existing cell-state models, but to strengthen them through more explicit attention to the tissue interface at which cell–cell interaction, metabolite distribution, stromal organization, and immune accessibility are observed together.

## Why the invasive front deserves a separate biological interpretation

Single-cell and spatial technologies have refined the classification of malignant, stromal, and immune states in HCC, while also showing that the biological relevance of these states depends on their spatial position and neighboring-cell context (7, 8). At the same time, recent work indicates that the biological significance of these states depends not only on what cells are present, but also on where they are located and with which neighboring populations they are associated (5, 9). The invasive front deserves particular attention because it is the region where tumor expansion, stromal activation, immune-cell positioning, and local metabolic gradients are most likely to be observed together.

Evidence from HCC and other solid tumors increasingly supports a spatial interpretation of tumor biology in which the invasive front and the adjoining tumor–stroma boundary serve as biologically informative units rather than simple geometric borders, with a multicellular ecosystem used here to emphasize the organized spatial coexistence of tumor, stromal, and immune populations. In colorectal cancer, Feng et al. showed that the spatial organization and immune status of the tumor–stroma boundary were closely associated with response to immune checkpoint blockade, and that fibroblast-associated structural barriers could distinguish immune-responsive from immune-restricted boundary states (10). In gastric cancer, Lee et al. further demonstrated that the tumor microenvironment can be dissected into spatially confined niches in which CCL2-positive fibroblasts and STAT3-activated macrophages participate in immunosuppressive crosstalk (11). In cervical cancer, Lin et al. showed that fibroblast states become more biologically informative when interpreted together with their spatial distribution and immune associations rather than as isolated stromal subsets (12). Together, these studies suggest that the invasive front is not simply a geometric border, but a spatial unit in which progression-associated cellular programs become regionally interpretable.

These observations also suggest that the invasive front may serve as a particularly useful analytical unit for HCC by providing a spatial context in which otherwise separate cellular programs become more biologically interpretable (4, 5, 13). At present, however, this perspective is best regarded as complementary to tumor-nest-based analysis rather than a replacement for it, and its added prognostic or therapeutic value will need to be tested directly in future HCC studies.

## A multicellular ecosystem at the tumor–stroma boundary

Among the spatial regions emerging from HCC studies, the tumor–CAF–macrophage interface appears especially informative because it is the local setting in which cellular interaction and metabolic interaction can be interpreted together. Measurements obtained from the tumor core or from immune cells distant from tumor nests remain valuable, but they do not always reflect the local tissue conditions under which malignant cells, fibroblasts, and macrophages exert their most direct reciprocal effects. By contrast, the interface at the invasive front more directly reflects the biological processes associated with progression because these cell populations are positioned within a shared local microenvironment. Although we refer to macrophages in a general sense throughout the manuscript, the spatial evidence currently available points more specifically to recurrent immunoregulatory macrophage states, particularly interface-associated populations such as SPP1-positive macrophages, rather than to the macrophage compartment as a whole.

Increasing evidence from other solid tumors suggests that the invasive front is often organized as a spatially restricted multicellular interface rather than a passive stromal border. In gastric cancer, localized crosstalk between CCL2-positive fibroblasts and STAT3-activated macrophages, as well as the adjacent distribution of GREM1-positive fibroblasts and SPP1-positive macrophages, further supports the idea that fibroblast–macrophage organization can define immunosuppressive interface states (11, 14). Comparable interface-like niches have also been reported in colorectal cancer liver metastasis and triple-negative breast cancer, where matrix-associated fibroblasts and SPP1-positive macrophages are spatially linked to immune restriction (15, 16). These observations suggest that the invasive front may be particularly informative because metabolic conditions, stromal structure, and cellular interaction are locally assembled within a common regional context, rather than being diffusely distributed across the tumor bed (17).

For HCC, a LEMCE-informed perspective is therefore useful not as a rigid label, but as a conceptual aid for interpreting the invasive front as a localized multicellular ecosystem.

## Metabolic interface as a unifying view of local tumor progression

We suggest that the concept of a metabolic interface is particularly useful for interpreting these observations. This concept emphasizes the interface itself and recognizes that multiple biochemical processes may contribute to local immunoregulatory conditions at the invasive front (3, 18).

Current evidence from HCC and other solid tumors supports several components of this view. Beyond HCC, pan-cancer analyses integrating single-cell and spatial transcriptomics have shown that stromal programs acquire additional biological meaning when interpreted together with spatial position and neighborhood structure, rather than through transcriptional identity alone (19, 20). Studies of tumor boundaries in breast cancer and colorectal cancer similarly indicate that malignant and non-malignant cells at these interfaces display distinct spatial features associated with progression, immune evasion, and treatment response (10, 19). In gastric cancer, immunosuppressive crosstalk between fibroblasts and macrophages has also been mapped to localized tumor niches rather than diffuse stromal compartments (11). In parallel, recent work on SPP1-positive macrophages and on the macrophage–ECM–CAF axis suggests that recurrent myeloid, matrix, and stromal programs may contribute to interface-associated immune restriction across tumor types (21).

This metabolic-interface perspective also clarifies why spatial analysis can add information beyond conventional compartment-based assessment. A glycolytic signal in the tumor core does not by itself indicate how fibroblasts or immune cells are positioned relative to that metabolic activity (5). Likewise, an exhausted immune phenotype detected in a distant immune region does not fully reveal the local stromal or metabolic conditions with which it is associated (4). By contrast, analysis of the interface makes it possible to examine tumor-cell metabolism, fibroblast activation, macrophage phenotype, matrix structure, and lymphocyte accessibility within the same tissue field. Restricted CD8-positive T-cell access is considered mainly a functional readout of interface organization and accessibility, rather than the defining cellular core of the interface itself. This is consistent with the broader concept that metabolic conditions within the tumor microenvironment reshape T-cell function, including through lipid metabolic adaptation, thereby linking local nutrient context to immune competence (22).

Recent technological development provides an additional reason to focus on the interface. Current spatial transcriptomic, proteomic, metabolomic, and multiplex imaging platforms have made it possible to identify cellular neighborhoods, functional niches, and interface-associated gradients with increasing fidelity, bringing important aspects of invasive-front biology into view (23–26). At the same time, many of these methods rely on fixed tissues or single sampling time points and therefore provide highly informative spatial snapshots of an evolving process. Rather than contrasting static and dynamic methods, these advances are better viewed as a continuum of technical development. Snapshot-based approaches define the relevant spatial structures, whereas longitudinal and spatiotemporal approaches can begin to show how those structures form, persist, or change under therapeutic pressure. For this reason, the next stage of interface research may be best understood as a progression from static spatial description to spatiotemporal interpretation.

As an additional descriptive phrase, some interface conditions may be viewed as forming a local metabolic shield, particularly when metabolite accumulation and stromal organization are associated with reduced T-cell access or function (1, 27). However, this should remain a subsidiary description of one possible interface phenotype rather than the main conceptual focus. The primary emphasis should remain on the metabolic interface itself and on the need to characterize its composition, variability, and biological significance with progressively more integrative spatial and temporal methods.

## Discussion

A metabolic-interface framework may have several implications for HCC research and clinical investigation. First, it may help refine biomarker development. Biomarkers derived solely from tumor-intrinsic expression or overall immune-cell abundance may not fully capture the localized tissue conditions associated with progression or treatment response (25, 28). A spatially defined tumor–CAF–macrophage interface may integrate fibroblast state, macrophage state, metabolite distribution, extracellular matrix organization, and lymphocyte accessibility, and may therefore provide particularly useful information for patient stratification.

Second, this perspective may help organize combination-therapy strategies more rationally. If biologically relevant processes are concentrated at specific invasive-front interfaces, then therapeutic design may benefit from addressing these local regions more explicitly rather than considering tumor cells, fibroblasts, and immune cells only as separate targets (7, 18). In HCC, CAF-FAP inhibition has been reported to enhance the antitumor effect of anti-PD-1 therapy in orthotopic models (2). Similarly, response or resistance after triple-combination therapy was associated with distinct spatial remodeling at the invasive margin, including redistribution of CD8-positive effector T cells away from immunosuppressive niches and enrichment of resistance-associated stromal and endothelial populations (28). Consistent with this, blockade of Arf1-mediated lipid metabolism has been shown to promote cytotoxic T-cell infiltration into tumors through a CCL5-dependent mechanism and to enhance the efficacy of PD-1 blockade, illustrating how tumor metabolic rewiring may directly modify immune accessibility (29). From a translational perspective, evaluation of such an interface may also depend on the available specimen type, including resection tissue, biopsy material, or post-treatment samples, which should be taken into account in future studies.

Third, this framework may also be useful across HCC etiologies. Spatial proteomic studies of NASH-associated HCC have shown multicellular immune interactions that differ from other HCC contexts, indicating that disease etiology can shape local tissue organization (30). Reviews of spatial liver biology and hepatobiliary tumor research likewise emphasize that tissue-contextual information can improve clinical interpretation and translational study design in liver disease and liver cancer (13, 26). This raises an important question: whether tumor–CAF–macrophage metabolic interfaces can be reproducibly defined across viral, alcohol-related, and metabolic dysfunction-associated HCC, and whether their composition or clinical relevance differs by etiology, stage, or treatment setting.

Future work could use orthotopic, carcinogen-induced, or genetically engineered mouse models combined with spatial and longitudinal readouts to examine how this interface develops, persists, and changes during therapy. Particularly informative next steps will include reproducible spatial co-localization across independent HCC cohorts, interface-specific metabolite enrichment, treatment-response associations, and perturbation studies showing altered immune accessibility. Integration of spatial metabolomics, multiplex imaging, and longitudinal analysis of invasive-front remodeling will be especially important for determining whether this proposed interface has practical value for biomarker development and therapeutic stratification. Recent spatial multi-omics studies suggest that the invasive front in HCC is a biologically informative region in which local metabolic conditions, stromal remodeling, and immune regulation can be interpreted together. We therefore propose that the tumor–CAF–macrophage metabolic interface may represent a useful conceptual framework of HCC precision oncology, as it may provide an integrated basis for biological interpretation, biomarker development, and therapeutic stratification.
